# Diphtheria, Croup and Tracheotomy

**Published:** 1877-12

**Authors:** 


### DIPHTHERIA, CROUP, AND TRACHEOTOMY.

M. Revilliod has reached the following conclusions:

Diphtheria is an acute specific malady, and characterized anatomically by peculiar false membranes in the respiratory tract. It manifests itself by variable lesions—at one time a benign form in which the symptoms depend solely upon a local lesion, at other times a malignant one, where there is an exhibition of general infection. A nosological distinction between croupal and diphtheritic affections, based upon pathological anatomy, does not conform to clinical experience. These two types belong to one and the same source, because—1, between them all sorts of intermediate forms are to be seen, both in local lesions and in general phenomena; and 2, they develop in the same epedimic, under the influence of the same *contagium;* and 3, often immediately succeed one another in the same individual. Like every infectious disease, the endemic diphtheria of cities is epidemic in the country.

The mortality by diphtheritic infection is greater in the cities than in the country, in the hospitals than in the cities, in the large hospitals than in the small. It varies just as the infective force, according to the period and the country. Diphtheria distinguishes itself from other virulent maladies by the special receptivity which certain families offer, in virtue of which brothers and sisters are often attacked successively under conditions of time and place which do not admit any question of contagion. There is no specific against diphtheria. In the third stage, tracheotomy is indicated whatever be the conditions of age, constitution, complication, or asphyxia. Two-fifths should recover. But success depends upon subsequent attention and the degree of the diphtheritic infection. One of the most frequent causes of death after tracheotomy is a trouble in the innervation of the pulmonary apparatus, and is really an extension of the paralysis of other parts, as shown by the expiring dyspnœa, anæsthesis of the thachea, and nutritive disorders of the lungs.—*La France Medicale*, 80, 1877. *Medical Record.*

